# Sickle Cell Hemoglobin “Drugged” with Cyclic Peptides Is Aggregation Incompetent

**DOI:** 10.1021/acs.jpcb.4c03805

**Published:** 2024-08-29

**Authors:** N. Galamba

**Affiliations:** Biosystems and Integrative Sciences Institute, Faculdade de Ciências da Universidade de Lisboa, Edifício C8, Campo Grande, 1749-016 Lisboa, Portugal

## Abstract

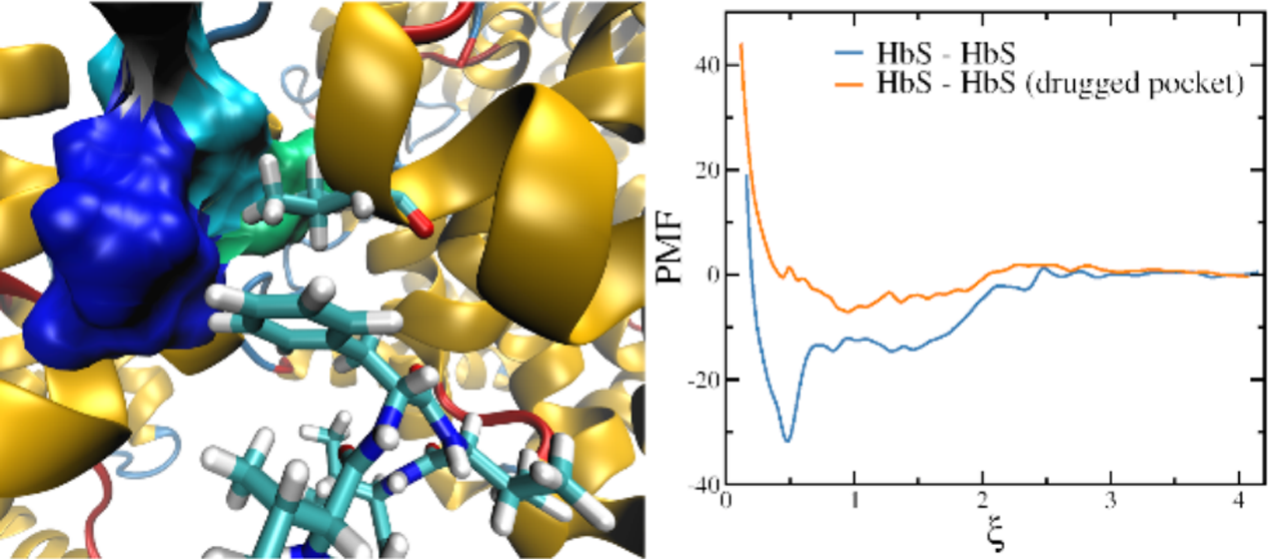

Sickle cell disease (SCD) is a monogenic blood disorder associated with a mutation in the hemoglobin subunit β gene encoding for the β-globin of normal adult hemoglobin (HbA). This mutation transcribes into a Glu-β6 → Val-β6 substitution in the β-globins, inducing the polymerization of this hemoglobin form (HbS) when in the T-state. Despite advances in stem cell and gene therapy, and the recent approval of a new antisickling drug, therapeutic limitations persist. Herein, we demonstrate through molecular dynamics and umbrella sampling, that (unrestrained) blockage of the hydrophobic pocket involved in the lateral contact of the HbS fibers by 5-mer cyclic peptides, recently proposed as SCD aggregation inhibitors (NetoV.; J. Med. Chem.2023, 66, 16062–1607437988411 10.1021/acs.jmedchem.3c01484), is enough to turn the dimerization of HbS thermodynamically unfavorable. Among these potential drugs, some exhibit an estimated pocket abandonment probability of around 15–20% within the simulations’ time frame, and an impressive specificity toward the mutated Val-β6. Additionally, we show that the dimerization can be thermodynamically unfavored by blocking a nearby region while the pocket remains vacant. These results are compared with curcumin, an antisickling molecule and a pan-assay interference compound, with a good binding affinity for different proteins and protein domains. Our results confirm the potential of some of these cyclic peptides as antisickling drug candidates to reduce the concentration of aggregation-competent HbS.

## Introduction

Sickle cell disease (SCD) is a missense genetic proteinopathy that manifests through the aggregation of the deoxygenated or T-state mutated hemoglobin (deoxy-HbS) into helical fibers that distort the erythrocytes’ shape. The single nucleotide change from adenine (A) to thymine (T) in the β-globin gene results in the substitution of a glutamic acid for a valine at the sixth position of the β-globins of normal adult hemoglobin, HbA.^1−6^ This substitution reduces the solubility^6^ of HbS, compared to HbA, prompting the aggregation of deoxygenated HbS (deoxy-HbS) into seven double stranded helical fibers, stretching and stiffening the erythrocytes.^6−16^ This morphological alteration results in abnormal adherence to endothelium cells, disrupting microcirculation (i.e., vaso-occlusion), and, thereby, insufficient oxygen delivery to tissues.^17,18^ HbS polymerization involves the formation of a lateral contact where the mutated residue (Val-β_2_6) lodges in a hydrophobic pocket of a neighbor HbS molecule. Weaker, mutation unrelated, axial contacts, are also involved in fiber growth.^19−21^

Although hematopoietic stem cell transplantation^22^ and gene therapy^23^ can cure SCD, both treatments have several limitations, including economic, and are not expected to become widely available in the near future, in countries where the disease has a high prevalence.^15,16,24−26^ Additionally, drugs such as hydroxyurea (aka hydroxycarbamide) or voxelotor, an allosteric modulator recently approved, aimed at stabilizing the nonpolymerizing relaxed form (R-state) of HbS, have limitations regarding their efficacy.^16,27,28^ Hydroxyurea, although the primary treatment for SCD since its approval in the late 1990s is not always effective.^29,30^ Henry et al.,^28^ in turn, recently showed that although Voxelotor significantly reduces sickling, oxygen delivery to tissues is offset by increased hemoglobin O_2_ affinity. A novel small molecule, named GBT021601, that increases HbS-oxygen affinity, with higher levels of Hb occupancy than Voxelotor, was also recently reported.^31^

Whereas these drugs result in a sickling decrease, their mode of action (MOA) does not involve the blocking of the lateral and/or axial contacts in the fibers. A drug that binds either to the hydrophobic pocket, the mutated Val-β6, or any other pivotal domain, underpinning the lateral contact in the fibers, should, in principle, inhibit or delay nucleation and, therefore, fiber formation. Nonetheless, there is no evidence that blocking this pocket is enough to deem the process thermodynamically unfavorable, since a larger HbS contact surface area is involved and electrostatic interactions connected with the absence of Glu-β6 also play an important role.^14,32−34^ Furthermore, while many small molecules with in vitro antisickling activity were discovered, their exact MOA proved difficult to establish. Several therapeutic strategies have been devised over the years in addition to the blockage of the direct contacts in the HbS fibers.^15,16,25,35−37^ These include the decrease of the intracellular HbS concentration, the decrease of the concentration of the allosteric effector, 2,3-diphosphoglycerate, increasing the solubility of HbS, and, among the most explored, the shift of the allosteric equilibrium toward the R-state of HbS.

A high-throughput assay (over 12,000 compounds) of the Scripps ReFRAME drug repurposing library, exploring all of the above MOAs, was recently reported.^30^ The sickling times of the compounds were assessed by following deoxygenation to 0% of red cells from sickle trait individuals and 106 antisickling compounds acting through some of the above MOAs were found. These and other antisickling potential drugs, including peptides, have been recently reviewed.^36,37^

Additionally, several 5-mer CPs, tailored designed to block the hydrophobic pocket and/or peripheral amino acids, were shown by our group to exhibit long residence times, low binding free energies (∼−30 kJ/mol), and a moderate to good specificity even at a (1:1) HbS/CP ratio.^38^ Furthermore, some CPs also exhibited some affinity toward the mutated Val-β6.

CPs are promising alternatives to small molecules and biologics in diseases associated with intracellular protein–protein aggregation due to their ability to bind to flat surfaces.^39−42^ Although HbS has a well-defined pocket, opposite, for instance, to intrinsically disordered proteins, implicated in neurodegenerative diseases, this is relatively shallow, and protein-CP binding could, therefore, be enhanced, compared to small molecules. Nonetheless, the proposed CPs do not distinguish from small molecules by their size, but rather by their tailor-design to bind either to the pocket or nearby amino acids shown to be involved in salt bridges (mostly Lys-Asp and Lys-Glu)^33,34,43^ with the neighbor HbS; their small size, and the formation of backbone intramolecular hydrogen-bonds,^38^ in turn, should favor membrane permeability.^40^ The CPs were found to bind orthogonally to the pocket, pointing one to two side chains to the pocket (generally a single Val or a Phe) while the remaining side chains interacted with pocket-neighbor amino acids and/or protruded to the solvent.^38^

Here, we investigated, through molecular dynamics and umbrella sampling,^44−46^ the aggregation of deoxy-HbS when the most promising cyclic peptides (CPs) from this previous study,^38^ occupy the hydrophobic pocket. An aspartic acid CP (DDDDD) that readily migrates to a nearby region,^38^ was also investigated, to assess the possibility of inhibiting dimerization when the pocket remains free. Additionally, curcumin, recently found to have antisickling activity by Metaferia et al.,^30^ was studied, to assess dimerization inhibition when the pocket is occupied by a different class of molecule (i.e., a polyphenol). Whereas the MOA of curcumin concerning its antisickling activity is unknown, this molecule has a good binding affinity for different proteins and protein domains, granting it the reputation of a pan-assay interference compound (PAINS),^47,48^ and, therefore, was chosen for comparison purposes.

## Methods

**Figure 1:**
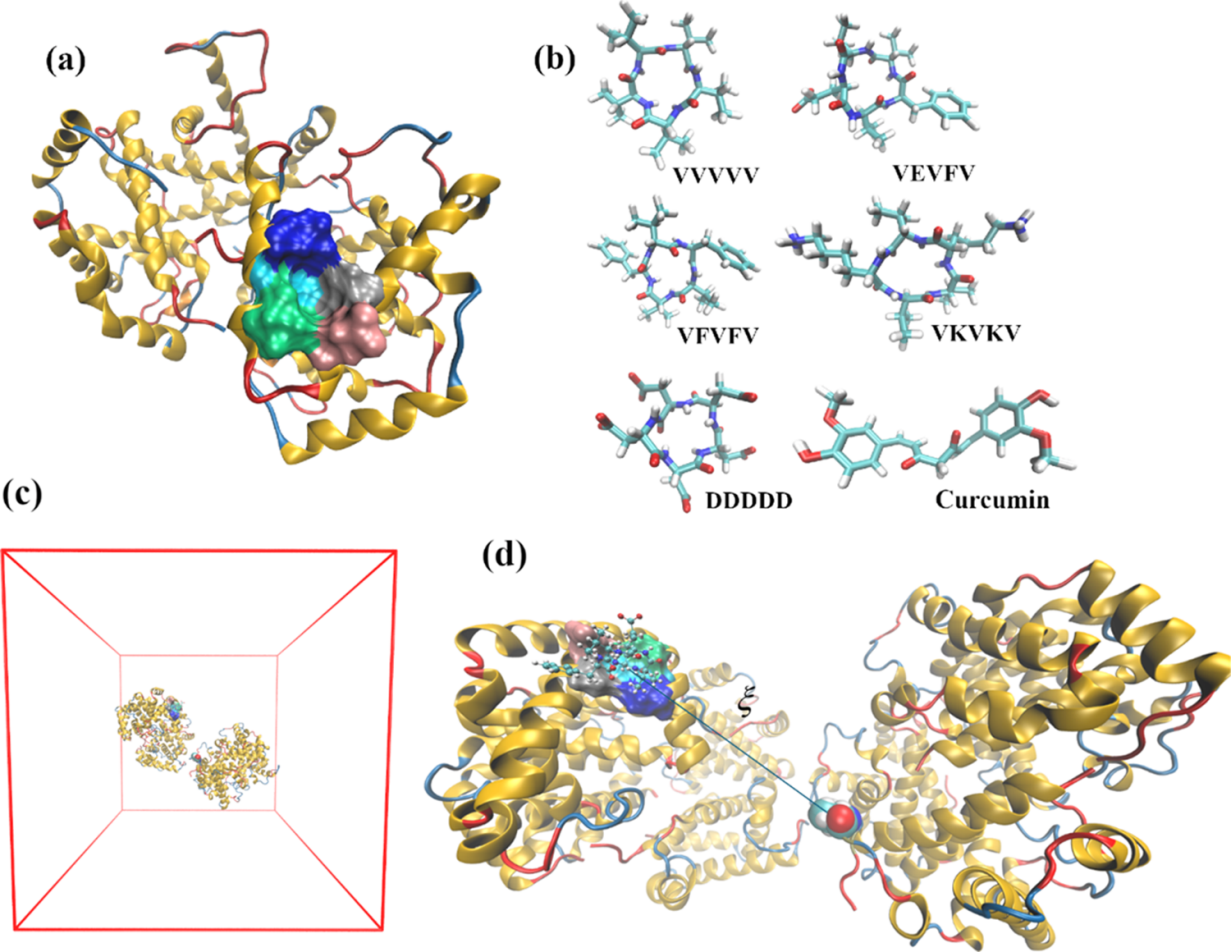
(a) HbS pocket (surface) where Val-β_2_6 is inserted in the HbS fibers. The pocket is defined by Ala70 (gray), Asp73 (pink), Thr84 (green), Phe85 (light blue), and Leu88 (dark blue). (b) The potential drugs studied in this work, namely, VVVVV (neutral), VEVFV (charge −1*e*), VFVFV (neutral), VKVKV (charge +2), DDDDD (charge −5*e*), and curcumin. (c) The starting production MD box with the HbS molecules used to calculate the window for a reaction coordinate value of 4 nm. (d) A close-up view of the 2 HbS in (c) showing a representation of the reaction coordinate; the pocket, on the left-hand-side HbS-1 is “drugged” with VEVFV; Val-β_2_6 is represented as magnified van der Waals spheres on the right-hand-side HbS-2.

Molecular dynamics (MD) of a deoxy-HbS dimer (2HbS) in 0.1 M NaCl aqueous solutions with and without different CPs or curcumin, were performed at 310 K and 0.1 MPa in a cubic box with periodic boundary conditions, using the program GROMACS, version 2021.2. The time averaged box side lengths was ∼15.8 nm and the 2HbS was solvated by ∼125,000 water molecules, for every system. The crystal structure of the dimer (pdb code:^14^2HBS) was used to generate the starting configurations of each system. HbS is comprised of 2α subunits (α-globin), each with 141 amino acids and a Heme group, and 2β subunits (β-globin), with 146 amino acids and a Heme group each. Each HbS has two mutated Val-β6, and two pockets, located at the β_1_-globin and β_2_-globin. The lateral contact in the HbS fibers involves the pocket in the β_1_-globin and the Val6-β_2_ of a neighbor HbS. Here, the pocket was defined by five amino acids in the β_1_-globin, namely, Ala-β_1_70, Phe-β_1_85, and Leu-β_1_88 (hydrophobic), and, Thr-β_1_84 and Asp-β_1_73 (hydrophilic), peripheral to the pocket^9−14^ (see Figure 1a).

The following 5-mer synthetic CPs were studied: VVVVV, VEVFV, VFVFV, VKVKV, and DDDDD. The first four peptides were found to be among the most promising in our previous study,^38^ concerning their specificity and residence time in the hydrophobic pocket (see Figure 1b). The aspartic acid CP (i.e., DDDDD) was chosen because this was found to migrate from the pocket to a nearby region,^38^ thus allowing studying whether HbS aggregation can be abrogated even when the pocket is always vacant. This is a highly charged peptide (*Q =* −5*e*) that can form salt bridges with HbS, potentially destabilizing HbS-HbS salt bridges.^33,34,43^ The peptides were cyclized through addition of a covalent bond between the C- and N-termini; thus, except for DDDDD this bond was formed between the C- and N-termini of Val amino acids.

The CPs, including DDDDD, as well as curcumin, were placed next to the pocket at time zero. The starting conformation was generated from the 2HbS crystal structure through steered MD, using a spring constant of 1000 kJ mol^–1^nm^–2^ and a pull rate of 0.01 nm ps^–1^. A conformation was chosen where enough space was available to insert the different molecules. For all CPs, except for DDDDD, one or two amino acid side chains were placed in the interior of the pocket. For curcumin, the two O–CH_3_ and one OH group were placed in the pocket, whereas for DDDDD a side chain was placed next to Thr84, as this geometry was observed in our previous study, among other structures.^38^ The starting geometries are displayed in Figure S1.

The windows at the different reaction coordinate values were then generated during the equilibration period. Thus, the CPs and curcumin were free to leave the pocket as the two HbS monomers were pulled apart via the umbrella (harmonic) potential, during the initial steps of equilibration. This is to be contrasted with a more biased approach, not followed here, where the molecules would be inserted in the pocket at time zero for each of the pre-equilibrated reaction coordinate configurations. The latter approach was not used, since we found that the CPs and curcumin remained in the pocket in most windows, during the equilibration period.

The HbS and CPs were modeled with the CHARMM36 force field, whereas water was described by the mTIP3P (i.e., CHARMM modified TIP3P) model.^49,50^ This water model differs from TIP3P in that H atoms have Lennard-Jones interactions, namely, σ_H_ = 0.04 nm and ε_H_ = 0.1925 kJ/mol. However, no differences were observed, between results obtained with TIP3P and mTIP3P.^38^

We calculated the potential of mean force (PMF), aka aggregation free energy profile, for the HbS dimer, when the pocket was vacant, and when a single molecule of the above drug candidates was placed next to the hydrophobic pocket of the HbS-1 (HbS “acceptor”) monomer, at time zero. The PMFs^51,52^ were computed through umbrella sampling.^44−46^ The reaction coordinate, ξ, was chosen to be the distance between the center of mass (COM) of the pocket at the β_1_-globin of HbS-1 and the COM of Val-β_2_6 of HbS-2 (see Figure 1c,d). The PMF is given (up to a constant *C*) by^46,53,54^
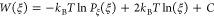
1where *P*_ξ_ is the probability distribution along the reaction coordinate ξ
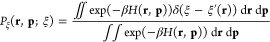
2*H* is the Hamiltonian, β = 1/*k*_B_*T*, *k*_B_ is the Boltzmann constant, **r** and **p**, are the Cartesian coordinates and the linear momenta of the particles, respectively, and the denominator is the partition function. The second term in eq 1 accounts for the transformation between Cartesian coordinates to the internal reaction coordinate (i.e., the pocket-Val-β_2_6 distance).^53^ This is obtained from the respective Jacobian (−ξ^2^ sin θ) and accounts for the increasing sampling volume with ξ in spherical polar coordinates. This term is, therefore, an entropic correction to the free energy along ξ.

In umbrella sampling a biased (*b*) probability distribution along ξ is calculated by adding a bias potential to the Hamiltonian, restraining the sampling along ξ
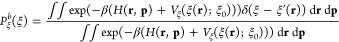
3A harmonic potential with a spring constant, *K*_ξ_, of 1000 kJ mol^–1^ nm^–2^ was used to restrain ξ
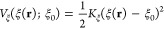
4and a spacing of 0.05 nm between windows (umbrellas) was used. The PMF for a window *i* is given by
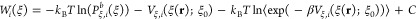
5The third term on the right-hand-side is an ensemble average, commonly represented by *F*_*i*_, that accounts for the free energy shift in window *i* due to the bias potential. This was estimated self-consistently through the weighted histogram analysis method^55^ (WHAM) to obtain the unbiased PMF. The PMFs were then shifted to zero at large separation distances (i.e.,  was used to define *C*) and the Bayesian bootstrap method^56^ using 200 bootstraps was used to estimate the errors.

The umbrella sampling trajectories (80 windows) were performed, respectively, for 60 ns for the 2HbS, and 30 ns for the “drugged” 2HbS systems, after steepest descent energy minimization, a 100 ps equilibration in the NVT ensemble, and a 10 ns equilibration in the NpT ensemble. The PMFs obtained from analysis of the first 30 ns and last 30 ns for “undrugged” 2HbS are nearly similar, indicating that 30 ns is enough to estimate the PMF (see Figure S2). We stress that no restraints were imposed on the molecules even during the equilibration period. This means that these were free to leave the pocket at any of the 80 windows.

The temperature (*T*) and pressure (*p*) were controlled with the Nosé–Hoover thermostat^57,58^ and the Parrinello–Rahman barostat.^59^ Electrostatic interactions were computed via the particle-mesh Ewald (PME) method.^60^ A cutoff of 1 nm was used for nonbonded van der Waals and for the PME real space electrostatic interactions. The equations of motion were solved with the Verlet leapfrog algorithm with a 2 fs time-step.

Additionally, 1 μs long, MD (3 replicates) of a single monomer of HbS and five molecules of the CP VEVFV were carried out. The CP molecules were randomly inserted in the solvent at time zero. These simulations were performed to confirm the specificity of this CP toward the mutated Val-β6, noted in the HbS-CP contact maps previously reported.^38^

We also performed an ordinary (unbiased) MD, 300 ns long, of the dimer (2HbS), to assess the CHARMM36 stability of the dimer. The root mean squared deviation (RMSD) was compared with those for the monomer of deoxy-HbS and normal human deoxy-HbA (pdb code:^61^2DN2) and are given in Figure S3. The RMSD of the dimer remains below 0.8 nm whereas those of the monomers remain below 0.6 nm, indicating that the dimer is stable and differences relative to the crystal are moderate.

## Results and Discussion

**Figure 2:**
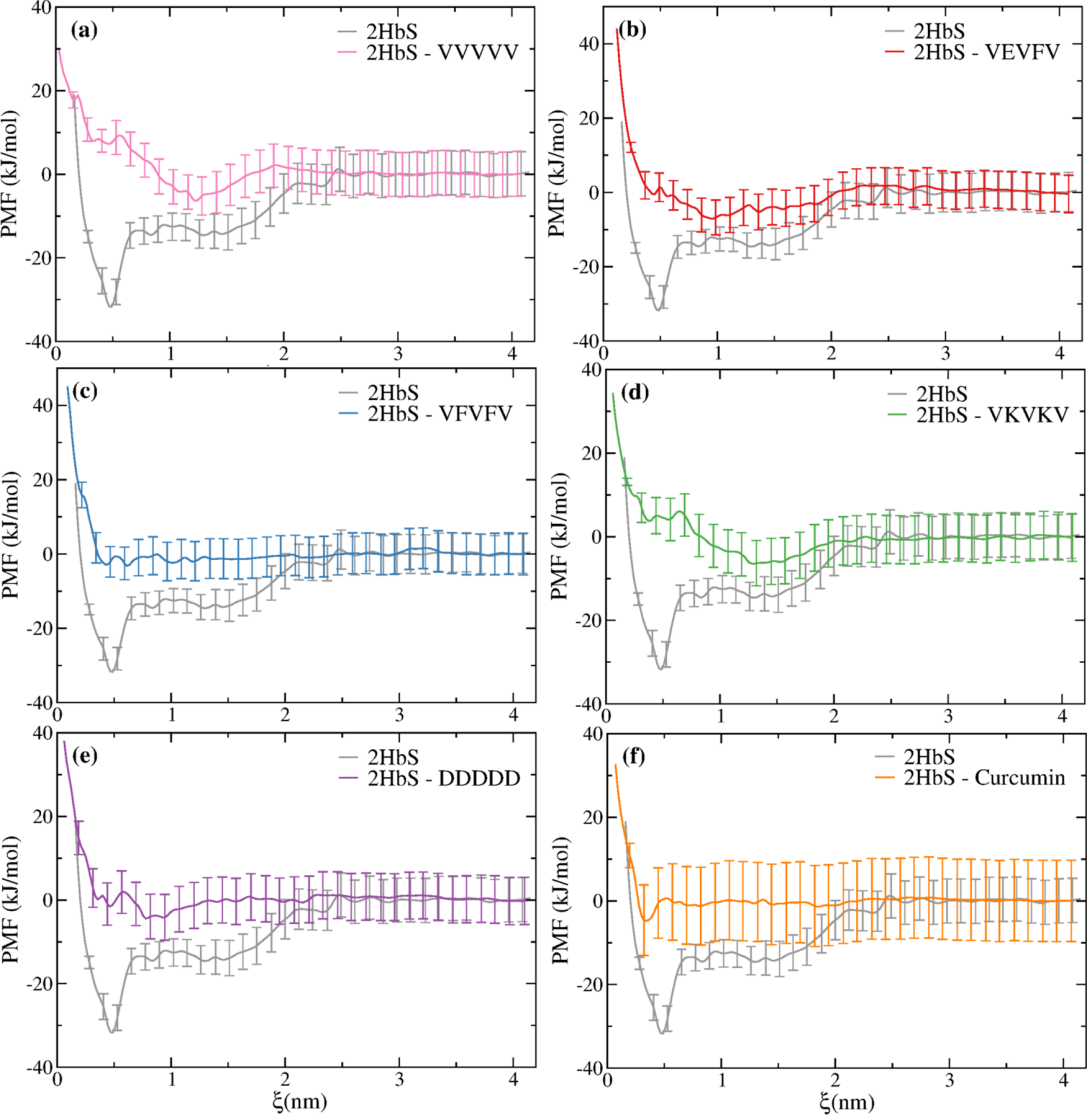
Potential of mean force calculated along the pocket-Val-β_2_6 distance, ξ, for the dimer “undrugged” (2HbS) and with the pocket of the HbS-1 (“acceptor”) “drugged” at time zero with a single molecule of (a) VVVVV, (b) VEVFV, (c) VFVFV, (d) VKVKV, (e) DDDDD, and (f) curcumin.

We begin our discussion by analyzing the potential of mean force (PMF) of 2HbS in the different systems (Figure 2). A dimerization free energy around −32 kJ mol^–1^ is found for the “undrugged” HbS dimer. We note that this value is higher than the value of ∼−57 kJ/mol^–1^ found from a one-dimensional PMF, although with a different force field (GROMOS54A7)^62^ and reaction coordinate.^33^ This is not unexpected because our reaction coordinate is more restrictive than the distance between the COM of HbS-1 and HbS-2, or one of its components,^33^ assuming the latter are similar within the simulations time frame, due to inertia;^33^ no linear relationship was found here between ξ and the HbS-1-HbS-2 COM distance (see Figure S4). We also note that the reaction coordinate used here allows using a significantly smaller MD box. The reason is that the reaction coordinate involves two surface groups instead of the HbS-HbS COM-COM distance. Since the geometry of HbS is more of a spheroid than of a sphere, and the proteins are free to rotate, some geometries at the same COM-COM distance involve much stronger protein–protein interactions, than others, due to a greater proximity between HbS-HbS surface amino acids. This results in a convergence of the PMF at a large value of ξ (>6.5 nm).^33,34^ For a HbS-HbS surface–surface reaction coordinate, this convergence occurs at much shorter distances (∼2.5 nm; see Figure 2) since ξ is imposed upon surface amino acids. Although less restrictive, a COM-COM reaction coordinate is not required for our purposes, since comparison is performed between the PMFs with and without drug candidates.

The binding free energy assessed here for the dimer, is nearly the same as that found between HbS and both, VVVVV and VEVFV, for a reaction coordinate defined as the distance between the COM of the pocket and the COM of the CPs.^38^

Figure 2a–f show that, although exhibiting different profiles, all CPs and curcumin reduce the aggregation propensity of HbS. For some CPs, namely, VVVVV, VEVFV, and VKVKV, a small minimum appears around ξ = 1.2 nm, broadly corresponding to the second (solvent separated) minimum of the PMF of the “undrugged” dimer. For others, such as VFVFV, DDDDD, and curcumin, such a minimum is less evident.

**Figure 3:**
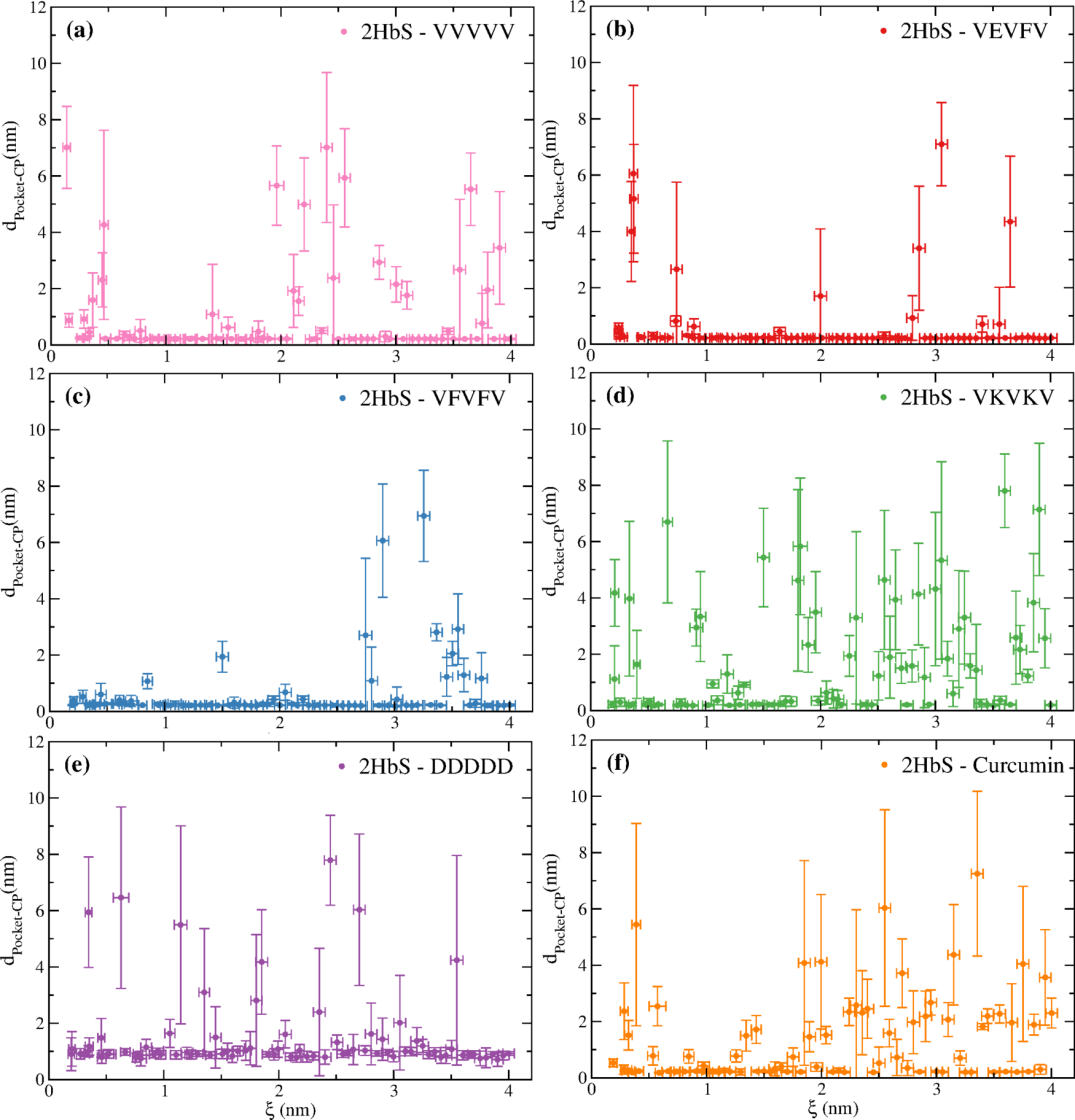
Average minimum distance between the pocket in HbS and (a) VVVVV, (b) VEVFV, (c) VFVFV, (d) VKVKV, (e) DDDDD, and (f) curcumin, for each of the 80 windows. Notice that DDDDD, although placed next to the pocket at time zero, readily diffuses to a neighbor region in all the windows. Points with large vertical error bars (distance standard deviations) are windows where the molecule left the pocket at some time, along the trajectories, resulting in large average minimum distances between the pocket and the CPs or curcumin. Horizontal error bars are reaction coordinate standard deviations for each window within the imposed harmonic restraint (see eq 4).

Figure 3a–f shows the average minimum distance between the pocket and the different molecules for the 80 windows (i.e., reaction coordinate distances). For some CPs, the number of windows where the molecules abandoned the pocket is especially small. Thus, for VEVFV and VFVFV the CPs only abandoned the pocket in about 15–20% of the windows. The highest probabilities of pocket abandonment are observed for VKVKV and curcumin (∼50%). Furthermore, the PMF of the latter shows a small minimum at the same position as the minimum found for “undrugged” 2HbS (Figure 2f).

Figure S5 compares the same PMFs of Figure 2, computed over the last 10 ns of the umbrella sampling trajectories. This shows that some PMFs start to converge to that of the “undrugged” 2HbS, with respect to the second (solvent separated) minimum, as more windows with the pocket or the interface region “undrugged” dominate in this time frame. For curcumin a more pronounced first minimum is visible (∼−10 kJ/mol).

We stress that the most promising CPs found in our previous work^38^ were VVVVV, VFVFV, VEVFV, and to a less extent VEVEV and VKVKV. These results indicate, therefore, that not only VVVVV, VFVFV, and VEVFV display long residence times next to the pocket and a moderate to high specificity for the pocket,^38^ but they are also able to turn the dimerization process thermodynamically unfavorable.

**Figure 4:**
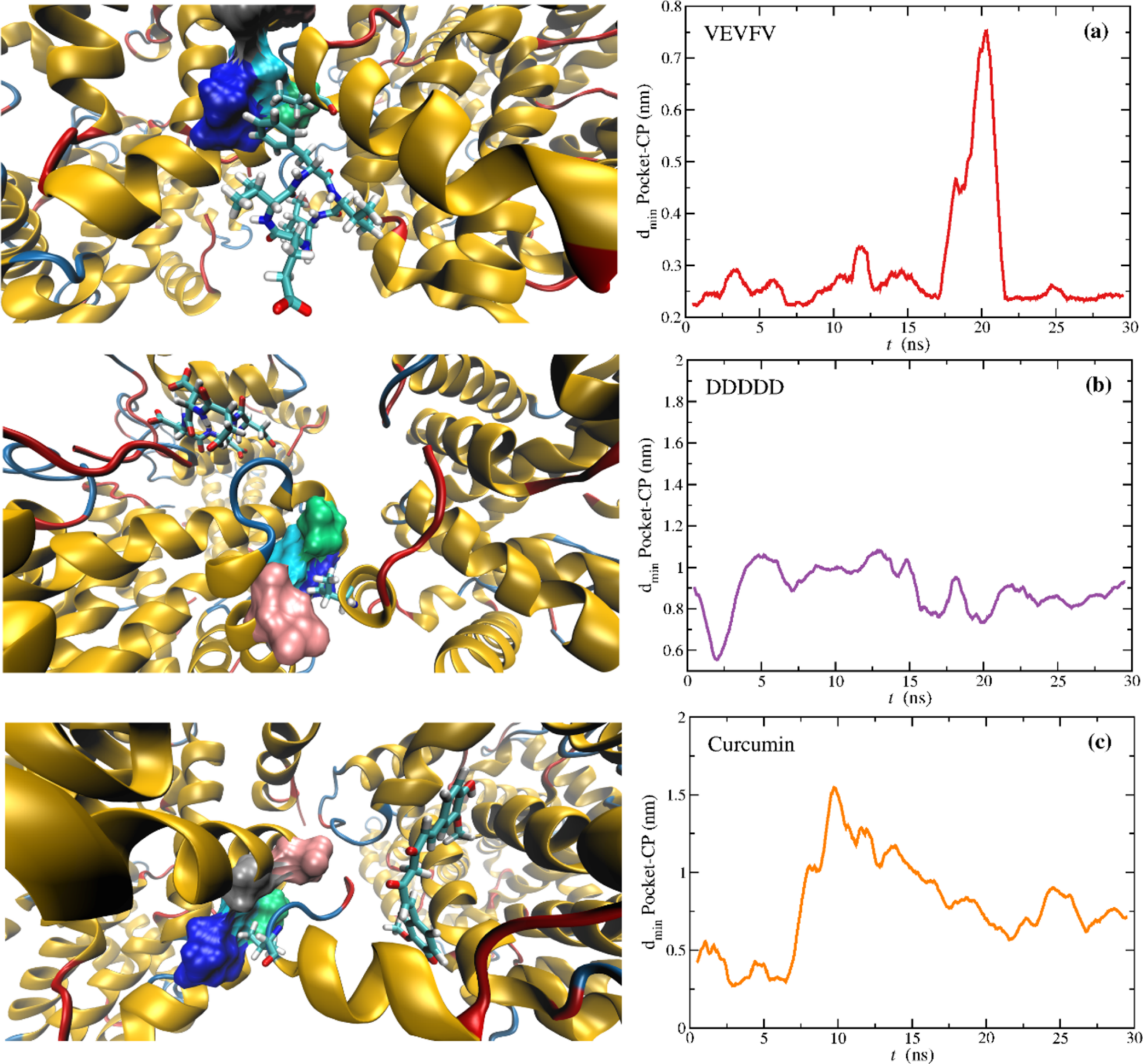
Last MD snapshot of the umbrella sampling trajectories for the window ξ = 0.5 nm, showing a zoomed image around the pocket and the moving average of the pocket-CP minimum distance along the trajectory for (a) VEVFV, (b) DDDDD, and (c) Curcumin. The pocket is represented as surface and the molecules and Val-β_2_6 from HbS-2 are represented in licorice. Please note the different scale of the *y*-axis between (a–c). A similar figure is given in Supporting Information (SI) for the other CPs (Figure S7).

Another noteworthy result concerns the behavior of DDDDD, for which a displacement from the pocket can be seen in every window, indicating that the CP migrated readily to a nearby region at ∼5–10 Å (see also Figure 4). Nonetheless, the probability of abandonment of this new region is quite small and the PMF is very similar to that found for VFVFV. A closer analysis of the regions where DDDDD is lodged shows that the main (positively) charged amino acids with which it interacts upon leaving the pocket are Lys-β_1_82, Lys-β_1_144, and Lys-β_1_131 from globin β_1_ and Lys-β_2_82, Val-β_2_1 (term-Val), His-β_2_2, His-β_2_77, and Lys-β_2_144 from globin β_2_.

Although DDDDD has limitations as a drug candidate, due to the high negative charge (−5*e*), including membrane permeation limitations, this compound demonstrates that it is possible to inhibit aggregation via the blockage of a different HbS-HbS contact, than the pocket. Thus, since DDDDD has a negative charge it may play a role similar to that of glutamate (Glu-β_2_6) in HbA, opposing the formation of salt bridges, as hypothesized before.^32−34^

Figure 3 also demonstrates that despite the restrain (i.e., harmonic potential) between the pocket and Val-β_2_6, the molecules can always leave the pocket, even at very short values of ξ. Thus, even for VKVKV and curcumin, the least compact molecules (see Figure 1), these can still diffuse away from the pocket at various windows at short distances. This is relevant because it demonstrates that our results are not biased, and that the molecules do not remain near the pocket at short values of ξ due to steric impediment associated with the harmonic restraint of the reaction coordinate. Furthermore, notice that the harmonic potential forces the Val-β_2_6 to enter the pocket at short distances, and, therefore, no molecule remains exactly inside the pocket. Thus, instead, some molecules preserve the contact with a specific residue of the pocket, even though they are prevented from occupying the inside of the pocket (Figure S6). This is the reason why no pocket abandonment (denoted by a large standard deviation in the *y*-axis) is always observed in Figure 3 for windows at small values of ξ.

Whereas Figure 3 provides an average picture of the behavior of these molecules in the different windows, this is characterized by fluctuations where the molecules move on the surface of both proteins, that is HbS-1 (“acceptor”) and HbS-2 (“donor”), near or away from the pocket. To illustrate some of these behaviors we show the last MD snapshot at ξ = 0.5 nm (see Figure 4), for 2HbS-VEVFV, 2HbS-DDDDD, and 2HbS-Curcumin. This is the ξ at which the PMF of “undrugged” HbS displays a minimum (contact pair).

Figure 4a shows the aromatic ring of VEVFV interacting with Leu-β_1_88 (dark blue) from the pocket, while Val-β_2_6 from HbS-2 is at 0.5 nm from the pocket COM. Noteworthy, the CP transiently drifted from the pocket at ∼20 ns, returning, however, in a small time frame. DDDDD is away from the pocket (Figure 4b), as previously discussed, whereas curcumin is in interaction with HbS-2, rather than with the pocket in HbS-1 (Figure 4c). Although these are three very different situations concerning HbS-ligand interactions, they all result in a weakening of the dimerization propensity. The other CPs exhibited a behavior similar to that found for VEVFV (see Figure S7), although for this particular window VVVVV abandoned the pocket during equilibration (Figure S7a). A possible reason for the enhanced performance of VEVFV is the fact that it can favorably interact with the pocket with four out of five amino acids, while the fifth residue (i.e., glutamate, E) can favorably interact with the solvent. Thus, this CP has a larger rotational freedom (entropic effect) next to the pocket, compared to other CPs, such as VKVKV, for which the residence time should also depend on the formation of salt bridges between the lysine residues with HbS-1 and/or HbS-2 (enthalpic effect).

**Figure 5:**
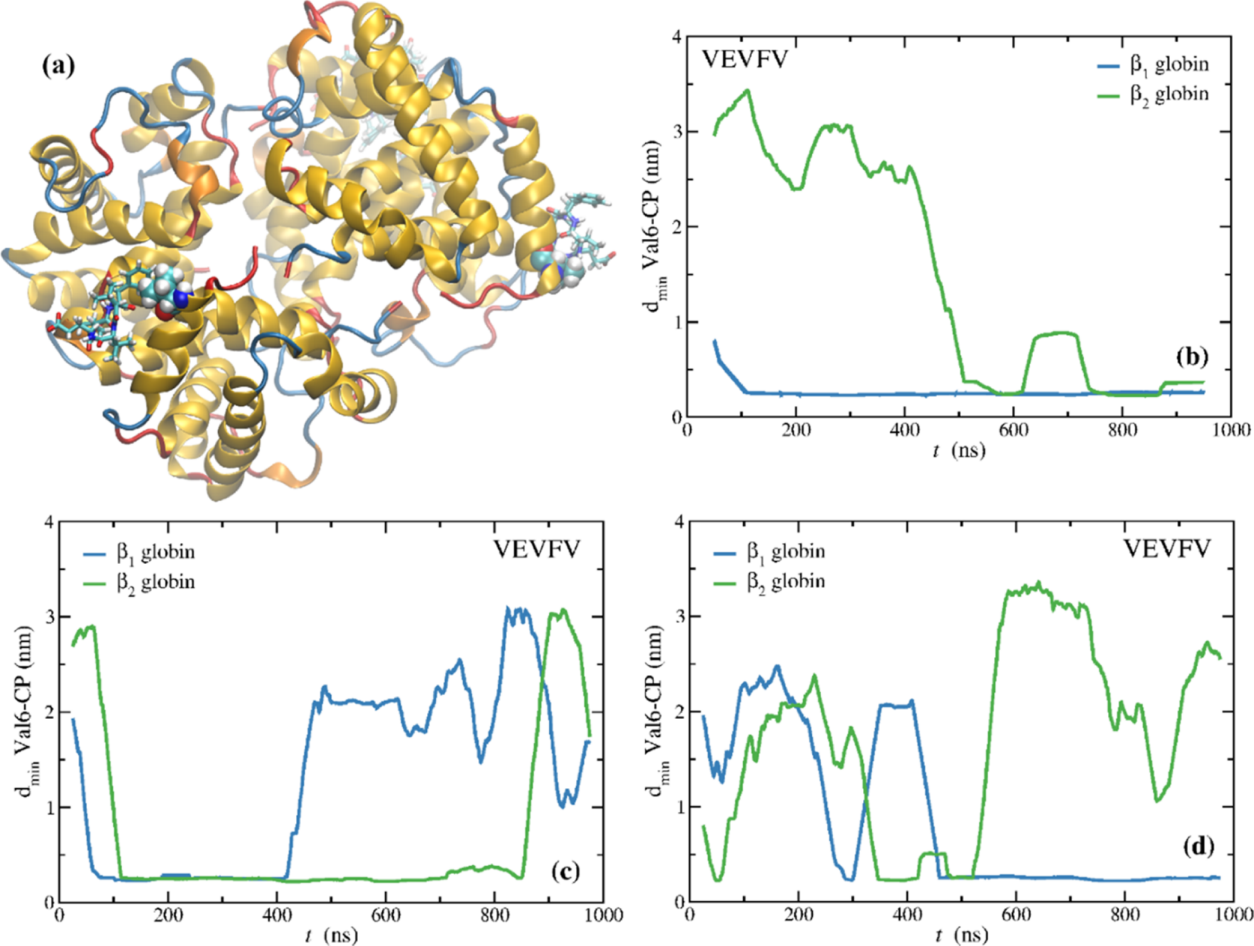
(a) MD snapshot from one of the replicates where both Val-β6 amino acids (van der Waals spheres) interact with VEVFV CPs (licorice); (b–d) moving averages of the minimum distance between the Val-β6 of globins β_1_ and β_2_, and the five CPs, for three MD replicates, 1 μs long.

Additionally, the CPs can also interact with the mutated Val-β6. Hence, we now discuss the specificity of VEVFV toward Val-β6. The efficiency of any potential aggregation inhibitor with this MOA relates to several factors, among which, its residence time, binding free energy, and specificity toward either the pocket or the Val-β6 mutated site. Neto et al.^38^ showed that VEVFV displayed the highest tendency to bind to the pocket, among the nine CPs studied. However, it was also noted that this CP interacted with Val-β6. To explore this further, we performed three MD, 1 μs long, of a monomer of HbS with five VEVFV CPs, randomly inserted in the solvent at time zero. Figure 5 shows the minimum distance between both Val-β6 residues (i.e., from globins β_1_ and β_2_) and the five CPs, throughout the trajectories. As can be seen in all trajectories at least one CP interacted with one of the Val-β6, and at some instances both Val-β6 amino acids were “blocked” by CPs (see Figure 5a). Furthermore, the residence times upon “binding” are larger than 500 ns in three instances.

These results are significant in that the same CP can bind to the pocket or to the mutated Val-β6, thus enhancing its potential ability to abrogate aggregation. We note that binding to the Val-β6 should be more probable than binding to the pocket, since this pocket already exists in the HbA, is relatively small, and nearly dehydrated, whereas the side chain of Val-β6 protrudes into the aqueous environment. Thus, aggregation should be mainly driven by the absence of Glu-β6 and the presence of Val-β6, and not so much by the existence of the pocket.^37^ Furthermore, as discussed by Neto et al., lasting binding events to the pocket require the approach of the CP with an orientation orthogonal to the pocket.^38^

## Conclusions

Although over 100 years have passed since SCD was discovered and 75 years since Pauling coined it, a molecular disease, there are still no effective and affordable treatments available. Therefore, the development of new antisickling molecules remains a pressing chemical and medical problem. Herein, we studied the impact of several promising cyclic peptides on the free energy of dimerization of a pair of HbS molecules, through molecular dynamics and umbrella sampling. Our results show that blockage of the hydrophobic pocket where Val-β_2_6 is lodged in the fibers is enough to turn the process thermodynamically unfavorable. Furthermore, a similar result can be seen for a CP (i.e., DDDDD) with little affinity for the pocket, that lodges instead in a region nearby, but outside the pocket. This suggests that aggregation can be inhibited by blocking electrostatic, rather than hydrophobic contacts in the dimer.^32−34,43^ Additionally, we confirmed that one CP, namely, VEVFV, has a significant specificity toward the mutated Val-β6, thus, increasing its potential efficiency as a protein aggregation inhibitor. Although in vitro and in vivo studies are necessary to probe the real potential of these CPs, including their membrane permeability, molecular simulations predict that some of these CPs might be viable antisickling molecules.

## Data Availability

All data are available in the manuscript or available from the corresponding author on reasonable request.

## Supporting Information Available

Starting protein-drug candidate geometries used in the umbrella sampling simulations (Figure S1); PMF for the 2HbS dimer from the first half and the last half of the umbrella sampling trajectories (Figure S2); moving average of the RMSD of 2HbS, HbS-1, HbS-2, and the monomers of HbS and HbA (Figure S3); HbS–HbS mean distances as a function of the mean values of the reaction coordinate (Figure S4); PMF calculated from the last 10 ns of the umbrella sampling trajectories (Figure S5); the last snapshot of the umbrella sampling trajectory of VEVFV for the window at the minimum value of the reaction coordinate, ξ = 0.05 nm (Figure S6), and the last snapshot of the umbrella sampling trajectories for the window ξ = 0.5 nm, and the moving average of the pocket-CP minimum distance, for VVVVV, VFVFV, and VKVKV (Figure S7) (PDF)

## References

[ref1] Herrick J. B. Peculiar Elongated and Sickle-Shaped Red Blood Corpuscles in a Case of Severe Anemia. Arch. Int. Med. 1910, VI (5), 517–521. 10.1001/archinte.1910.00050330050003. PMC2588723 11501714

[ref2] Pauling L.; Itano H. A.; Singer S. J.; Wells I. C. Sickle Cell Anemia, a Molecular Disease. Science 1949, 110 (2865), 543–548. 10.1126/science.110.2865.543. 15395398

[ref3] Ingram V. M. A Specific Chemical Difference Between the Globins of Normal Human and Sickle-Cell Anæmia Hæmoglobin. Nature 1956, 178 (4537), 792–794. 10.1038/178792a0. 13369537

[ref4] Ingram V. M. Gene Mutations in Human Hæmoglobin: The Chemical Difference Between Normal and Sickle Cell Hæmoglobin. Nature 1957, 180 (4581), 326–328. 10.1038/180326a0. 13464827

[ref5] Noguchi C. T.; Schechter A. N. Sickle Hemoglobin Polymerization in Solution and in Cells. Annu. Rev. Biophys. Biophys. Chem. 1985, 14 (1), 239–263. 10.1146/annurev.bb.14.060185.001323. 3890882

[ref6] Eaton W. A.; Hofrichter J. Sickle Cell Hemoglobin Polymerization. Adv. Protein Chem. 1990, 40, 63–279. 10.1016/S0065-3233(08)60287-9. 2195851

[ref7] Perutz M. F. Stereochemistry of Cooperative Effects in Haemoglobin: Haem–Haem Interaction and the Problem of Allostery. Nature 1970, 228 (5273), 726–734. 10.1038/228726a0. 5528785

[ref8] Edelstein S. J.; Telford J. N.; Crepeau R. H. Structure of Fibers of Sickle Cell Hemoglobin. Proc. Natl. Acad. Sci. U.S.A. 1973, 70 (4), 1104–1107. 10.1073/pnas.70.4.1104. 4123929 PMC433435

[ref9] Wishner B. C.; Ward K. B.; Lattman E. E.; Love W. E. Crystal Structure of Sickle-Cell Deoxyhemoglobin at 5 Å Resolution. J. Mol. Biol. 1975, 98 (1), 179–194. 10.1016/S0022-2836(75)80108-2. 1195378

[ref10] Dykes G.; Crepeau R. H.; Edelstein S. J. Three-Dimensional Reconstruction of the Fibres of Sickle Cell Haemoglobin. Nature 1978, 272 (5653), 506–510. 10.1038/272506a0. 692655

[ref11] Dykes G. W.; Crepeau R. H.; Edelstein S. J. Three-Dimensional Reconstruction of the 14-Filament Fibers of Hemoglobin S. J. Mol. Biol. 1979, 130 (4), 451–472. 10.1016/0022-2836(79)90434-0. 480359

[ref12] Padlan E. A.; Love W. E. Refined Crystal Structure of Deoxyhemoglobin S I. Restrained Least-Squares Refinement at 3.0–8 Resolution. J. Biol. Chem. 1985, 260 (14), 8272–8279. 10.1016/S0021-9258(17)39466-8. 4008491

[ref13] Padlan E. A.; Love W. A. Refined Crystal Structure of Deoxyhemoglobin S II. Molecular Interactions in the Crystal. J. Biol. Chem. 1985, 260 (14), 8280–8291. 10.1016/S0021-9258(17)39467-X. 2409085

[ref14] Harrington D. J.; Adachi K.; Royer W. E. The High Resolution Crystal Structure of Deoxyhemoglobin S. J. Mol. Biol. 1997, 272 (3), 398–407. 10.1006/jmbi.1997.1253. 9325099

[ref15] Eaton W. A.; Bunn H. F. Treating Sickle Cell Disease by Targeting HbS Polymerization. Blood 2017, 129 (20), 2719–2726. 10.1182/blood-2017-02-765891. 28385699 PMC5437829

[ref16] Tisdale J. F.; Thein S. L.; Eaton W. A. Treating Sickle Cell Anemia. Science 2020, 367 (6483), 1198–1199. 10.1126/science.aba3827. 32165573 PMC7299198

[ref17] Eaton W. A.; Hofrichter J. The Biophysics of Sickle Cell Hydroxyurea Therapy. Science 1995, 268 (5214), 1142–1143. 10.1126/science.7539154. 7539154

[ref18] Kato G. J.; Piel F. B.; Reid C. D.; Gaston M. H.; Ohene-Frempong K.; Krishnamurti L.; Smith W. R.; Panepinto J. A.; Weatherall D. J.; Costa F. F.; Vichinsky E. P. Sickle Cell Disease. Nat. Rev. Dis. Primer 2018, 4 (1), 18010 10.1038/nrdp.2018.10. 29542687

[ref19] Ferrone F. A.; Hofrichter J.; Eaton W. A. Kinetics of Sickle Hemoglobin Polymerization. I. Studies Using Temperature-Jump and Laser Photolysis Techniques. J. Mol. Biol. 1985, 183 (4), 591–610. 10.1016/0022-2836(85)90174-3. 4020872

[ref20] Ferrone F. A.; Hofrichter J.; Eaton W. A. Kinetics of Sickle Hemoglobin Polymerization. II. A Double Nucleation Mechanism. J. Mol. Biol. 1985, 183 (4), 611–631. 10.1016/0022-2836(85)90175-5. 4020873

[ref21] Wang Y.; Ferrone F. A. Dissecting the Energies That Stabilize Sickle Hemoglobin Polymers. Biophys. J. 2013, 105 (9), 2149–2156. 10.1016/j.bpj.2013.09.032. 24209860 PMC3824546

[ref22] Eapen M.; Brazauskas R.; Walters M. C.; Bernaudin F.; Bo-Subait K.; Fitzhugh C. D.; Hankins J. S.; Kanter J.; Meerpohl J. J.; Bolaños-Meade J.; et al. Effect of Donor Type and Conditioning Regimen Intensity on Allogeneic Transplantation Outcomes in Patients with Sickle Cell Disease: A Retrospective Multicentre, Cohort Study. Lancet Haematol. 2019, 6 (11), e585–e596. 10.1016/S2352-3026(19)30154-1. 31495699 PMC6813907

[ref23] Ribeil J.-A.; Hacein-Bey-Abina S.; Payen E.; Magnani A.; Semeraro M.; Magrin E.; Caccavelli L.; Neven B.; Bourget P.; El Nemer W.; et al. Gene Therapy in a Patient with Sickle Cell Disease. N. Engl. J. Med. 2017, 376 (9), 848–855. 10.1056/NEJMoa1609677. 28249145

[ref24] Kapoor S.; Little J. A.; Pecker L. H. Advances in the Treatment of Sickle Cell Disease. Mayo Clin. Proc. 2018, 93 (12), 1810–1824. 10.1016/j.mayocp.2018.08.001. 30414734

[ref25] Telen M. J.; Malik P.; Vercellotti G. M. Therapeutic Strategies for Sickle Cell Disease: Towards a Multi-Agent Approach. Nat. Rev. Drug Discovery 2019, 18 (2), 139–158. 10.1038/s41573-018-0003-2. 30514970 PMC6645400

[ref26] Cisneros G. S.; Thein S. L. Recent Advances in the Treatment of Sickle Cell Disease. Front. Physiol. 2020, 11, 435 10.3389/fphys.2020.00435. 32508672 PMC7252227

[ref27] de Montellano P. R. O. A New Step in the Treatment of Sickle Cell Disease: Published as Part of the *Biochemistry* Series “Biochemistry to Bedside.. Biochemistry 2018, 57 (5), 470–471. 10.1021/acs.biochem.7b00785. 29172465

[ref28] Henry E. R.; Metaferia B.; Li Q.; Harper J.; Best R. B.; Glass K. E.; Cellmer T.; Dunkelberger E. B.; Conrey A.; Thein S. L.; et al. Treatment of Sickle Cell Disease by Increasing Oxygen Affinity of Hemoglobin. Blood 2021, 138 (13), 1172–1181. 10.1182/blood.2021012070. 34197597 PMC8570057

[ref29] Rankine-Mullings A.; Reid M.; Soares D.; Taylor-Bryan C.; Wisdom-Phipps M.; Aldred K.; Latham T.; Schultz W. H.; Knight-Madden J.; Badaloo A.; et al. Hydroxycarbamide Treatment Reduces Transcranial Doppler Velocity in the Absence of Transfusion Support in Children with Sickle Cell Anaemia, Elevated Transcranial Doppler Velocity, and Cerebral Vasculopathy: The EXTEND Trial. Br. J. Hamaetol. 2021, 195 (4), 612–620. 10.1111/bjh.17698. 34291449

[ref30] Metaferia B.; Cellmer T.; Dunkelberger E. B.; Li Q.; Henry E. R.; Hofrichter J.; Staton D.; Hsieh M. M.; Conrey A. K.; Tisdale J. F.; et al. Phenotypic Screening of the ReFRAME Drug Repurposing Library to Discover New Drugs for Treating Sickle Cell Disease. Proc. Natl. Acad. Sci. U.S.A. 2022, 119 (40), e2210779119 10.1073/pnas.2210779119. 36161945 PMC9546543

[ref31] Dufu K.; Alt C.; Strutt S.; Partridge J.; Tang T.; Siu V.; Liao-Zou H.; Rademacher P.; Williams A. T.; Muller C. R.; et al. GBT021601 Improves Red Blood Cell Health and the Pathophysiology of Sickle Cell Disease in a Murine Model. Br. J. Hamaetol. 2023, 202 (1), 173–183. 10.1111/bjh.18771. 36960712

[ref32] Kuczera K.; Gao J.; Tidor B.; Karplus M. Free Energy of Sickling: A Simulation Analysis. Proc. Natl. Acad. Sci. U.S.A. 1990, 87 (21), 8481–8485. 10.1073/pnas.87.21.8481. 2236057 PMC54980

[ref33] Galamba N.; Pipolo S. On the Binding Free Energy and Molecular Origin of Sickle Cell Hemoglobin Aggregation. J. Phys. Chem. B 2018, 122 (30), 7475–7483. 10.1021/acs.jpcb.8b03708. 29995412

[ref34] Galamba N. On the Nonaggregation of Normal Adult Hemoglobin and the Aggregation of Sickle Cell Hemoglobin. J. Phys. Chem. B 2019, 123 (50), 10735–10745. 10.1021/acs.jpcb.9b09727. 31747289

[ref35] Dean J.; Schechter A. N. Sickle-Cell Anemia: Molecular and Cellular Bases of Therapeutic Approaches: (First of Three Parts). N. Engl. J. Med. 1978, 299 (14), 752–763. 10.1056/NEJM197810052991405. 357967

[ref36] Olubiyi O. O.; Olagunju M. O.; Strodel B. Rational Drug Design of Peptide-Based Therapies for Sickle Cell Disease. Molecules 2019, 24 (24), 4551 10.3390/molecules24244551. 31842406 PMC6943517

[ref37] Martins G. F.; Galamba N. Protein Aggregation-Inhibition: A Therapeutic Route from Parkinson’s Disease to Sickle Cell Anemia. Crit. Rev. Biochem. Mol. Biol. 2023, 58 (1), 50–80. 10.1080/10409238.2023.2201406. 37158748

[ref38] Neto V.; Victor B. L.; Galamba N. Cyclic Peptides as Aggregation Inhibitors for Sickle Cell Disease. J. Med. Chem. 2023, 66 (23), 16062–16074. 10.1021/acs.jmedchem.3c01484. 37988411

[ref39] Dougherty P. G.; Qian Z.; Pei D. Macrocycles as Protein–Protein Interaction Inhibitors. Biochem. J. 2017, 474 (7), 1109–1125. 10.1042/BCJ20160619. 28298556 PMC6511976

[ref40] Dougherty P. G.; Sahni A.; Pei D. Understanding Cell Penetration of Cyclic Peptides. Chem. Rev. 2019, 119 (17), 10241–10287. 10.1021/acs.chemrev.9b00008. 31083977 PMC6739158

[ref41] Cunningham A. D.; Qvit N.; Mochly-Rosen D. Peptides and Peptidomimetics as Regulators of Protein–Protein Interactions. Curr. Opin. Struct. Biol. 2017, 44, 59–66. 10.1016/j.sbi.2016.12.009. 28063303 PMC5496809

[ref42] Vinogradov A. A.; Yin Y.; Suga H. Macrocyclic Peptides as Drug Candidates: Recent Progress and Remaining Challenges. J. Am. Chem. Soc. 2019, 141 (10), 4167–4181. 10.1021/jacs.8b13178. 30768253

[ref43] Olagunju M. O.; Loschwitz J.; Olubiyi O. O.; Strodel B. Multiscale MD Simulations of Wild-type and Sickle Hemoglobin Aggregation. Proteins: Struct., Funct., Bioinf. 2022, 90 (11), 1811–1824. 10.1002/prot.26352. 35475513

[ref44] Torrie G. M.; Valleau J. P. Nonphysical Sampling Distributions in Monte Carlo Free-Energy Estimation: Umbrella Sampling. J. Comput. Phys. 1977, 23 (2), 187–199. 10.1016/0021-9991(77)90121-8.

[ref45] Torrie G. M.; Valleau J. P. Monte Carlo Free Energy Estimates Using Non-Boltzmann Sampling: Application to the Sub-Critical Lennard-Jones Fluid. Chem. Phys. Lett. 1974, 28 (4), 578–581. 10.1016/0009-2614(74)80109-0.

[ref46] Kästner J. Umbrella Sampling. WIREs Comput. Mol. Sci. 2011, 1 (6), 932–942. 10.1002/wcms.66.

[ref47] Baell J.; Walters M. A. Chemistry: Chemical Con Artists Foil Drug Discovery. Nature 2014, 513 (7519), 481–483. 10.1038/513481a. 25254460

[ref48] Young L. M.; Ashcroft A. E.; Radford S. E. Small Molecule Probes of Protein Aggregation. Curr. Opin. Chem. Biol. 2017, 39, 90–99. 10.1016/j.cbpa.2017.06.008. 28649012 PMC7617773

[ref49] Best R. B.; Zhu X.; Shim J.; Lopes P. E. M.; Mittal J.; Feig M.; MacKerell A. D. Optimization of the Additive CHARMM All-Atom Protein Force Field Targeting Improved Sampling of the Backbone ϕ, ψ and Side-Chain χ _1_ and χ _2_ Dihedral Angles. J. Chem. Theory Comput. 2012, 8 (9), 3257–3273. 10.1021/ct300400x. 23341755 PMC3549273

[ref50] Huang J.; MacKerell A. D. CHARMM36 All-Atom Additive Protein Force Field: Validation Based on Comparison to NMR Data. J. Comput. Chem. 2013, 34 (25), 2135–2145. 10.1002/jcc.23354. 23832629 PMC3800559

[ref51] Kirkwood J. G. Statistical Mechanics of Fluid Mixtures. J. Chem. Phys. 1935, 3 (5), 300–313. 10.1063/1.1749657.

[ref52] Chandler D. Introduction to Modern Statistical Mechanics; Oxford University Press: New York, 1987.

[ref53] Trzesniak D.; Kunz A. E.; Van Gunsteren W. F. A Comparison of Methods to Compute the Potential of Mean Force. ChemPhysChem 2007, 8 (1), 162–169. 10.1002/cphc.200600527. 17131434

[ref54] Roux B. The Calculation of the Potential of Mean Force Using Computer Simulations. Comput. Phys. Commun. 1995, 91 (1–3), 275–282. 10.1016/0010-4655(95)00053-I.

[ref55] Kumar S.; Rosenberg J. M.; Bouzida D.; Swendsen R. H.; Kollman P. A. The Weighted Histogram Analysis Method for Free-Energy Calculations on Biomolecules. I. The Method. J. Comput. Chem. 1992, 13 (8), 1011–1021. 10.1002/jcc.540130812.

[ref56] Hub J. S.; de Groot B. L.; van der Spoel D. G_wham—A Free Weighted Histogram Analysis Implementation Including Robust Error and Autocorrelation Estimates. J. Chem. Theory Comput. 2010, 6 (12), 3713–3720. 10.1021/ct100494z.

[ref57] Evans D. J.; Holian B. L. The Nose–Hoover Thermostat. J. Chem. Phys. 1985, 83 (8), 4069–4074. 10.1063/1.449071.

[ref58] Hoover W. G. Canonical Dynamics: Equilibrium Phase-Space Distributions. Phys. Rev. A 1985, 31 (3), 1695 10.1103/PhysRevA.31.1695. 9895674

[ref59] Parrinello M.; Rahman A. Polymorphic Transitions in Single Crystals: A New Molecular Dynamics Method. J. Appl. Phys. 1981, 52 (12), 7182–7190. 10.1063/1.328693.

[ref60] Essmann U.; Perera L.; Berkowitz M. L.; Darden T.; Lee H.; Pedersen L. G. A Smooth Particle Mesh Ewald Method. J. Chem. Phys. 1995, 103 (19), 8577–8593. 10.1063/1.470117.

[ref61] Park S.-Y.; Yokoyama T.; Shibayama N.; Shiro Y.; Tame J. R. H. 1.25 Å Resolution Crystal Structures of Human Haemoglobin in the Oxy, Deoxy and Carbonmonoxy Forms. J. Mol. Biol. 2006, 360 (3), 690–701. 10.1016/j.jmb.2006.05.036. 16765986

[ref62] Oostenbrink C.; Villa A.; Mark A. E.; Van Gunsteren W. F. A Biomolecular Force Field Based on the Free Enthalpy of Hydration and Solvation: The GROMOS Force-Field Parameter Sets 53A5 and 53A6. J. Comput. Chem. 2004, 25 (13), 1656–1676. 10.1002/jcc.20090. 15264259

