# Supplementary material for: Sickle Cell Hemoglobin “Drugged” with Cyclic Peptides Is Aggregation Incompetent
Source: J Phys Chem B. 2024 Aug 29;128(36):8662–71. doi: 10.1021/acs.jpcb.4c03805 (PMC11403655; doi:10.1021/acs.jpcb.4c03805)
Supplement: Supplementary file 1 — jp4c03805_si_001.pdf [file jp4c03805_si_001.pdf]

Sickle Cell Hemoglobin "Drugged" with Cyclic Peptides is Aggregation Incompetent

Supporting Information

N. Galamba[a,*]

[a] Biosystems and Integrative Sciences Institute, Faculdade de Ciências da Universidade de Lisboa, Edifício C8, Campo Grande, 1749-016 Lisboa, Portugal

[*]Corresponding author: njgalamba@fc.ul.pt

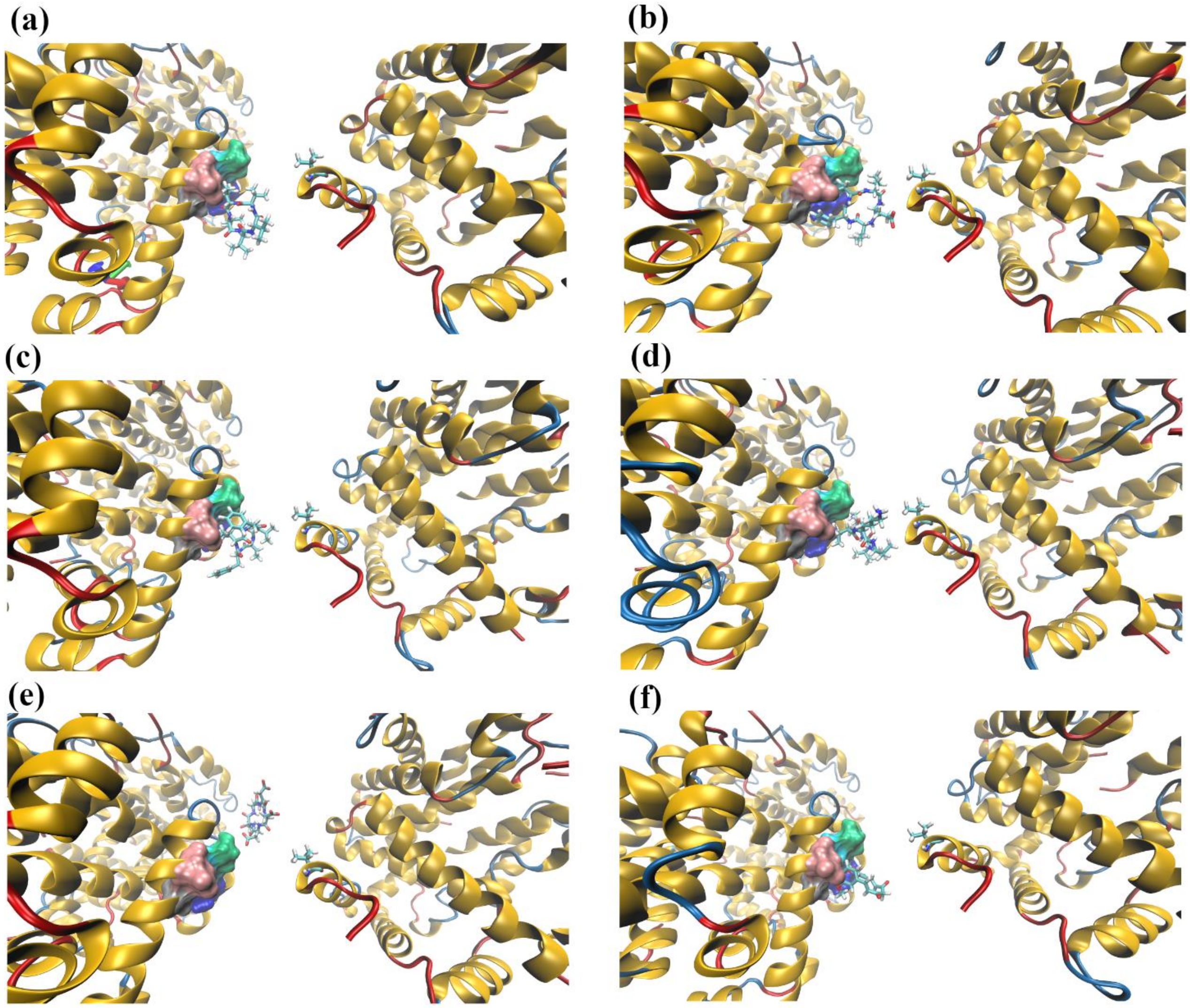

Figure S1 – Starting protein-drug candidate geometries used in the umbrella sampling simulations for (a) VVVVV, (b) VEVFV, (c) VFVFV, (d) VKVKV, (e) DDDDD, and (f) curcumin. The energy of the aqueous solution was minimized using the steepest descent method; water molecules are omitted for clarity.

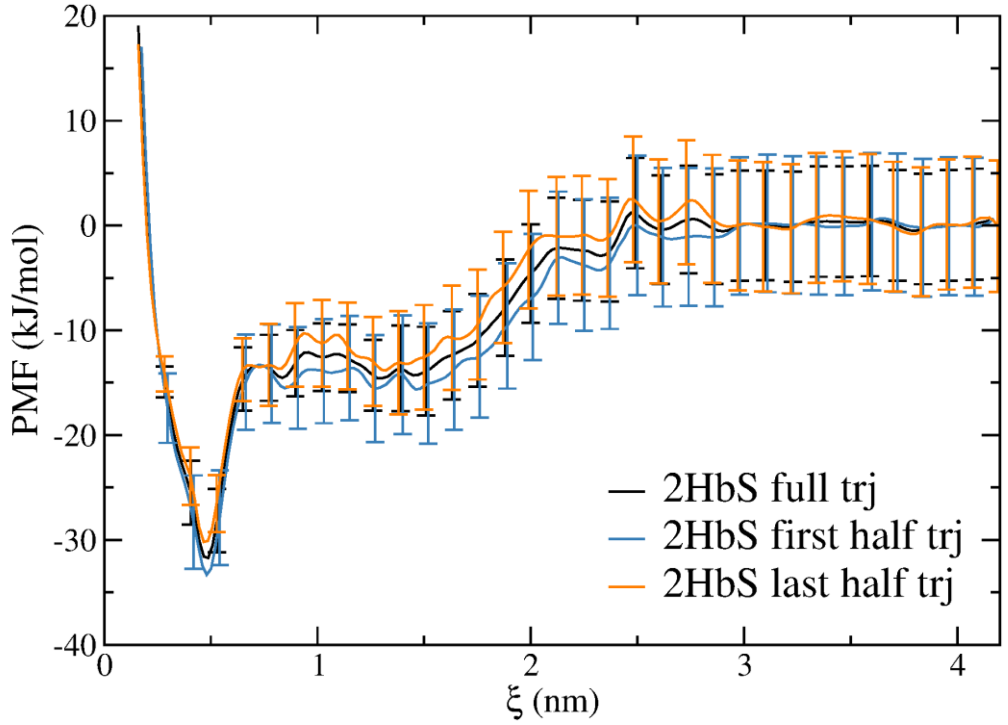

Figure S2 - Potential of mean force for the 2HbS dimer calculated along the pocket-Val6 distance for the dimer undrugged (2HbS). The PMF obtained from the full trajectory (60 ns) and the first and last 30 ns is depicted.

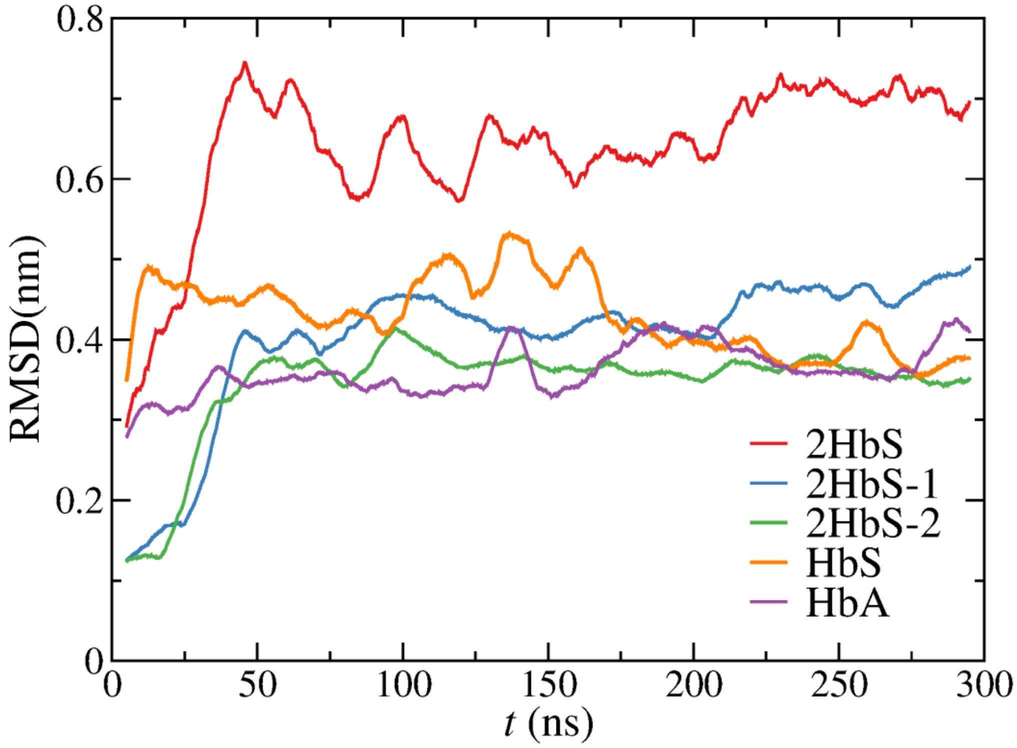

Figure S3 – Moving average of the RMSD of 2HbS, HbS-1 (HbS acceptor), HbS-2 (HbS donor), and the monomers of HbS and HbA.

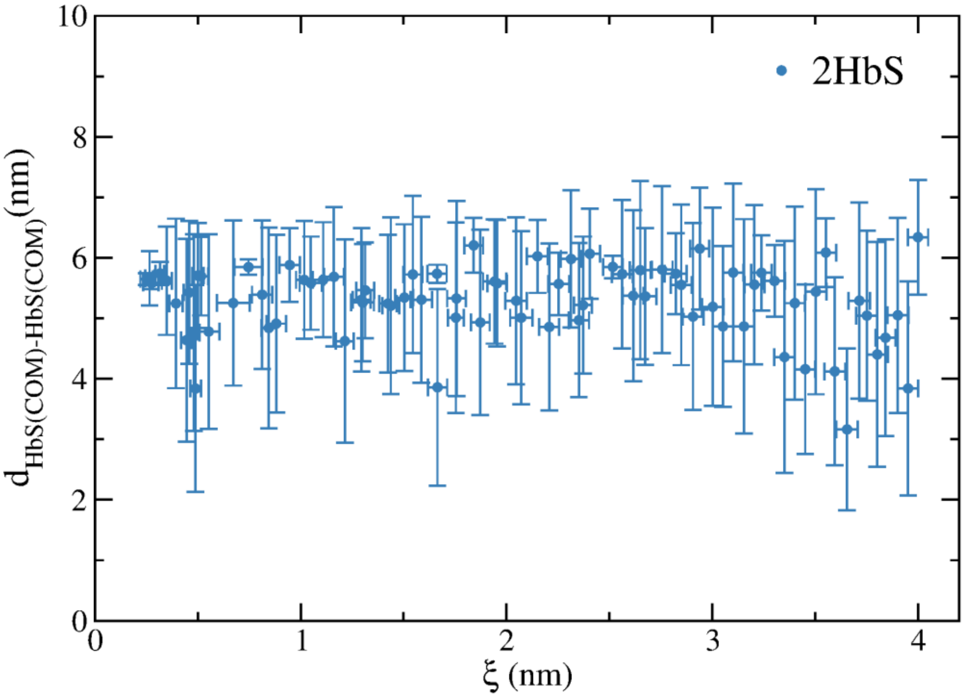

Figure S4 – HbS-1(COM)–HbS-2(COM) mean distances as a function of the mean values of the reaction coordinate computed from the umbrella sampling trajectories for the HbS dimer (undrugged). Error bars are standard deviations of the mean.

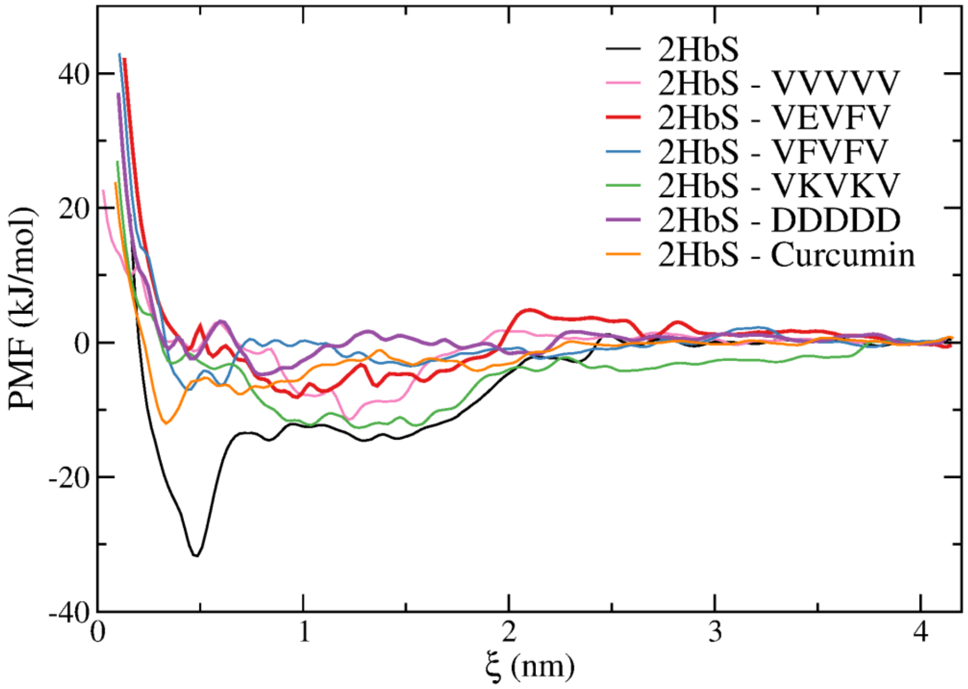

Figure S5 – PMF calculated from the last 10 ns of the umbrella sampling trajectories.

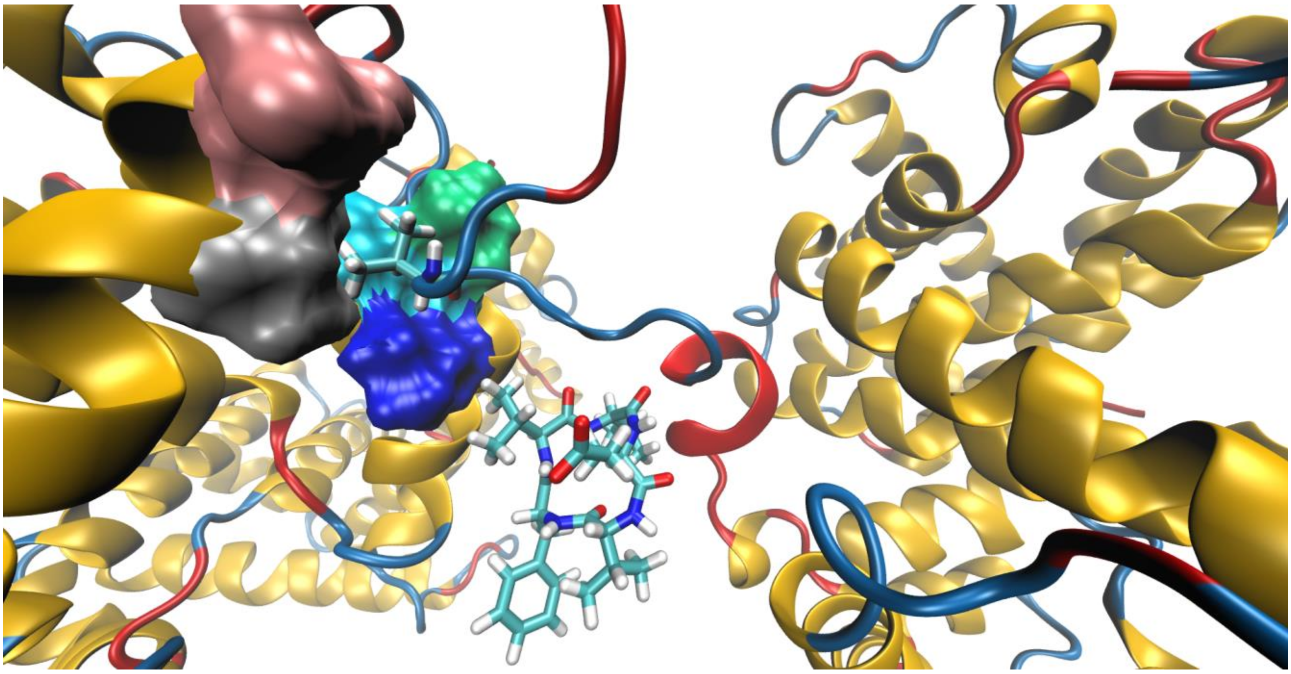

Figure S6 – The last snapshot of the umbrella sampling trajectory of VEVFV for the window at the minimum value of the reaction coordinate, ξ = 0.05 nm. Notice the pocket is occupied by Val whereas one Val side chain remains in close contact with Leu88.

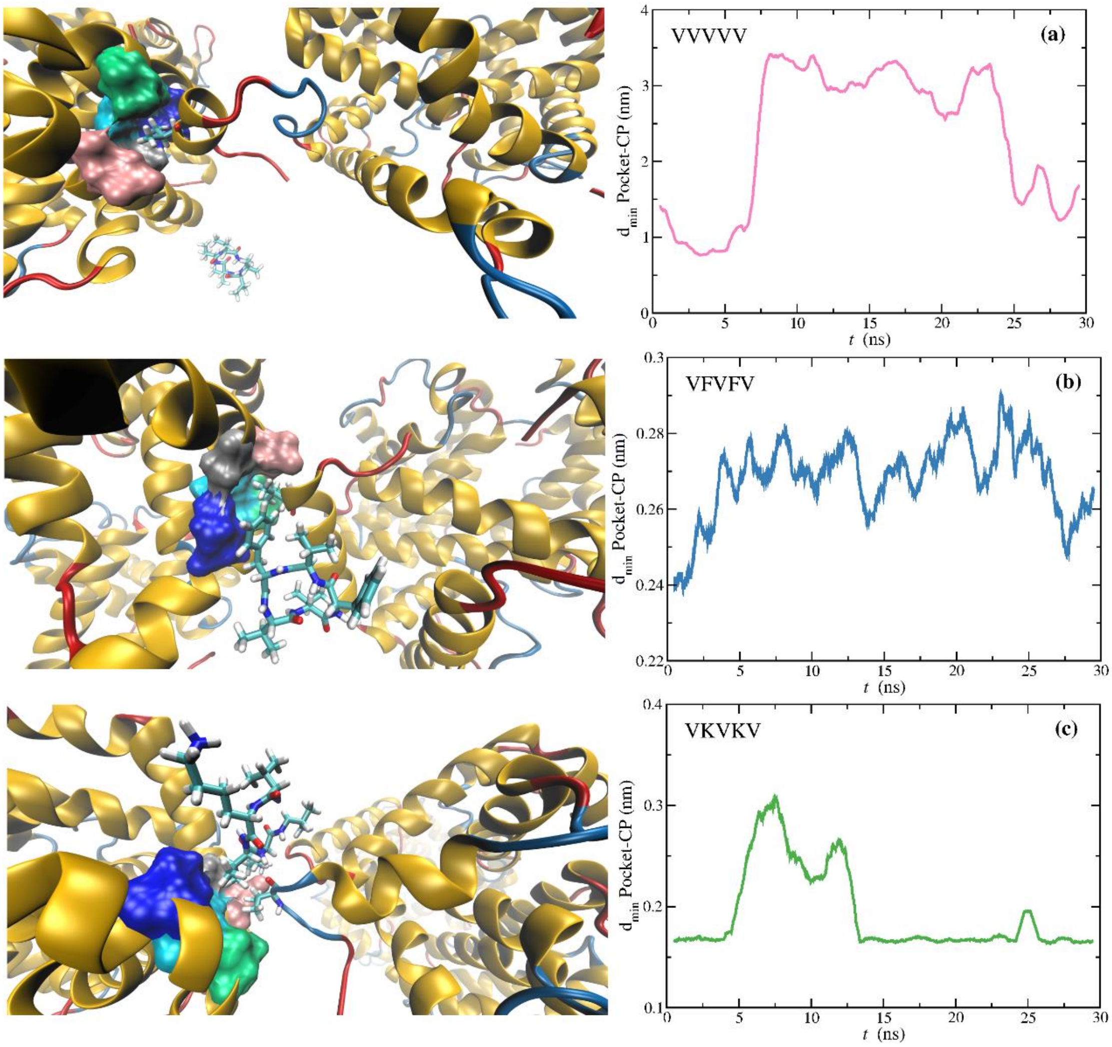

Figure S7 – The last snapshot of the umbrella sampling trajectories for the window ξ = 0.5 nm, showing a zoomed image around the pocket and the moving average of the pocket-CP minimum distance along the trajectory for **(a)** VVVVV, **(b)** VFVFV, and **(c)** VKVKV. The pocket is represented as surface and the drugs and Val-$\beta_2$6 from HbS-2 are represented in licorice. Please note the different scale of the *y*-axis between (a), (b), and (c).
